# Revealing isochoric water nucleation: a visual study

**DOI:** 10.1038/s41598-024-61053-y

**Published:** 2024-05-02

**Authors:** Ștefan-Ioan Câmpean, George-Andrei Beșchea, Maria-Bianca Tăbăcaru, Gabriel Năstase

**Affiliations:** Department of Building Services, Faculty of Civil Engineering, Transilvania University of Brasov, Brasov, Romania

**Keywords:** Chemical physics, Engineering, Chemical engineering

## Abstract

The phenomena of water freezing at constant volume, or isochoric, is becoming more and more fascinating. However, because the system is subjected to extremely high pressures, it is exceedingly challenging to investigate it visually. Fewer properties have been found visually up till now, but many have been found through other means. Nevertheless, we were able to design a reactor so that it could be observed through a microscope as water was frozen and thawed at constant volume, reaching temperatures as low as − 12 °C and pressures up to 129 MPa. In this study, we observed critical characteristics visually, focusing on the location of the ice nucleus, its shape, and dynamics. Phase transitions from liquid to solid state are essential mechanisms in the physical sciences. The creation of ice stands as the quintessential and pervasive example of nucleation, playing a central role in diverse disciplines such as geology, biology, aviation, and climate research.

## Introduction

Water nucleation is a fundamental phenomenon in the field of physical chemistry, representing the initial stages of phase transition from liquid to solid ice. In this phase transition, water molecules navigate a delicate balance between thermal motion and intermolecular forces, culminating in the initiation of a solid phase nucleus. The nucleation and growth of ice crystals are influenced by the change in Gibbs free energy^1,2^. The formation of ice nuclei and subsequent growth involve a reduction in free energy, driving the system towards a more stable state. The Clausius–Clapeyron equation ^3,4^ describes the relationship between temperature and pressure during phase transitions. It is relevant to water nucleation as changes in temperature and pressure can affect the rate and characteristics of ice nucleation. Kinetic factors^5^, such as the rate of molecular motion and collision frequencies, are crucial in determining the speed at which nucleation occurs. The Arrhenius equation ^6,7^ is often applied to describe the temperature dependence of reaction rates, including those associated with nucleation. Heterogeneous nucleation^8^ involves the presence of foreign particles or surfaces that can serve as nucleation sites, reducing the energy barrier for ice formation. The Kelvin equation ^9^ is relevant in the context of heterogeneous nucleation on foreign particles. In homogeneous nucleation, ice nuclei form spontaneously in the absence of foreign particles. Homogeneous nucleation^10^ is influenced by factors such as temperature, pressure, and the molecular structure of the liquid. Van’t Hoff's factor^11^ is relevant in solutions where solutes may influence the freezing point of water. It helps predict the change in freezing point depression based on the number of particles in solution.

Understanding the interplay of these laws and concepts is crucial for unravelling the complexities of water nucleation from liquid to solid ice under various conditions. Researchers employ these principles to model and predict ice formation in diverse settings, ranging from atmospheric processes to the controlled freezing of biological samples and the design of freezing processes in materials science.

The phase transition of liquid water to solid ice occurs when the temperature falls below 0 °C, under atmospheric and constant pressure. Under these specific conditions, the volume of water expands^12^ because of the ice crystals formation. The aqueous phase undergoes a complete transition into a solid state, forming ice. Same thermodynamics occur in constant volume conditions, except only a volume percentage of ice is formed, relative to the initial system volume (volume ice/initial volume)^12^. This is well known, and it’s dictated by the thermodynamic equilibrium phase diagram of water. The relation between the percentage volume of ice (IP%) and the phase transition temperature in an isochoric system, for water was theoretically investigated^13^. Investigating and understanding the intricacies of water nucleation below freezing temperatures is essential for elucidating the thermodynamic and kinetic mechanisms governing ice formation, with implications ranging from atmospheric processes^14^ to cryopreservation technologies^15,16^.

Reducing the temperature, extends life by lowering metabolism. However, biological matter is made primarily of water and since water freezes at 0 °C, major biological damage can be induced. Motivated by an interest in developing new cryopreservation technologies without the formation of ice crystals in a biological matter we developed a device where the thermodynamics and kinetic mechanisms governing ice formation in isochoric conditions can be visualised. An approximately similar device and a demonstration of its use in cryopreservation was for the first time introduced in a recent study of Professor Boris Rubinsky and his team at Berkeley University^17^. We replicated and optimized the isochoric (constant-volume) supercooling cryomicroscope (ISCM) as will be described in the “Materials and methods” section.

The phenomenon of phase transition has captivated researchers, serving not only as an intriguing academic challenge in condensed matter physics and statistical physics but also as a subject of technological interest^18^. Nucleation is often explained by the classical nucleation theory (CNT), wherein a nascent phase, referred to as emerging from the solution, occurs in a singular step^19,20^. The nucleation process is influenced by a multitude of factors, including temperature, pressure, impurities, and the presence of nucleation sites. The promotion of nucleation has been recognized through surface roughness. Indeed, it is a prevalent experimental procedure to intentionally scratch surfaces, inducing nucleation on irregularities like grooves and pits^21,22^.

At sub-zero temperatures, the transition from liquid to solid water typically begins with the formation of tiny ice nuclei, around which additional water molecules coalesce, ultimately giving rise to macroscopic ice crystals. The study of water freezing under different conditions, including isochoric scenarios where volume remains constant, provides valuable insights into the underlying mechanisms governing ice nucleation.

As researchers delve into the complexities of water nucleation leading to the formation of ice, they contribute not only to the fundamental understanding of physical processes but also to the development of technologies related to cryopreservation, ice formation control, and environmental monitoring.

Our goal in this study is purely to reveal the isochoric water nucleation evolution in an isochoric cryomicroscope. We see it as a new tool to observe the preservation of biological mater at subfreezing temperatures in isochoric conditions and for that we need to anticipate the thermodynamic and the kinetic mechanisms of nucleation in such systems.

This study sets the stage for delving into the intricate world of water nucleation, underscoring its importance across diverse scientific disciplines and applications. As researchers continue to explore the complexities of this phenomenon, new insights are emerging that contribute to our broader understanding of the physical properties of water and its role in shaping the world around us.

## Materials and methods

### Design of the partially transparent isochoric observation reactor

In the ice nucleation process, water molecules come together to form ice crystals. This typically occurs below freezing point under normal atmospheric pressure. When water is isochorically frozen, it results in a significant increase in pressure arresting the full ice formation within the system. At − 10 °C, the pressure can reach about 100 MPa, and it rises linearly to around 200 MPa at − 20 °C. When developing systems that require freezing operations at constant volume, engineers and scientists must take this into consideration to ensure the equipment's safety and integrity.

It can be difficult to design a system that allows you to see within an aqueous substance when it undergoes isochoric freezing, which can result in a considerable increase in pressure. To design the system, it was taken into consideration: transparent materials such as quartz, sapphire and certain high-strength glasses that can handle high-pressures, reinforcement for sustaining the transparent windows with strong and stable metals with high thermal transfer. Such equipment was designed in research about survival of HeLa cells in isochoric supercooling conditions^23^. In this study, ice preferentially forms across the sapphire windows of the reactor’s chamber due to the high heat conductivity of the sapphire lens relative to the stainless steel 316 chamber walls, making inspections inside the chamber impossible once nucleation has taken place^23^.

To overcome this barrier, it was chosen a material that was adequately durable yet had a higher heat transfer coefficient than sapphire. To force the ice nucleus form on the metal walls of the reactor, in this study we used aluminium 7075-T6 which has a thermal conductivity more than 3.5 times higher than that of sapphire, and great strength to withstand very high pressures inside the isochoric reactor.

**Figure 1:**
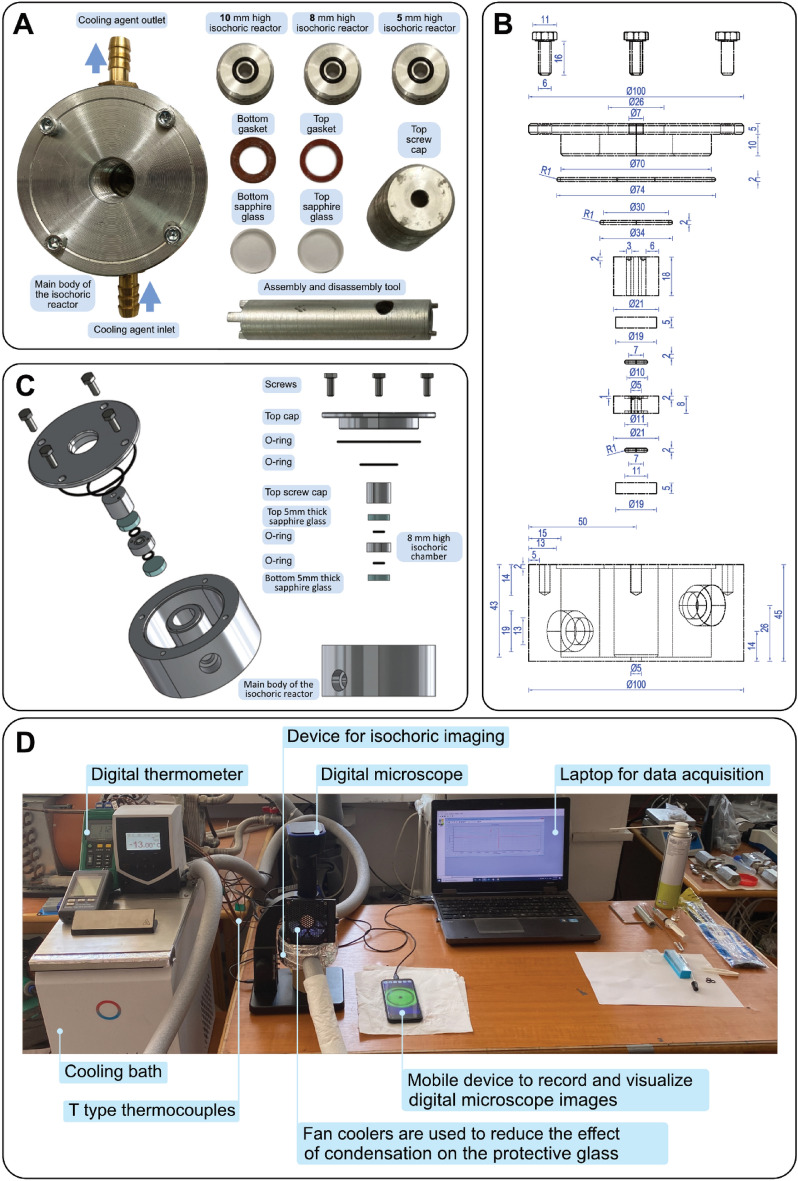
(**A**) The isochoric visualization device and all its components; (**B**) the schematic of the isochoric visualization device; (**C**) isometric (left) and front (right) view of exploded 3D computer model. (**D**) The complete setup for microscopic nucleation visualization.

In the paragraphs that follows, it will be provided a quick overview of the system used to observe the nucleation process and ice growth in isochoric conditions. Figure 1 shows the cylindrical apparatus that performs two essential functions: maintaining the stability of the isochoric chamber and uniformly cooling its walls with the coolant. The height of the apparatus is 50 mm and the outer diameter is 100 mm (Fig. 1).

**Figure 2:**
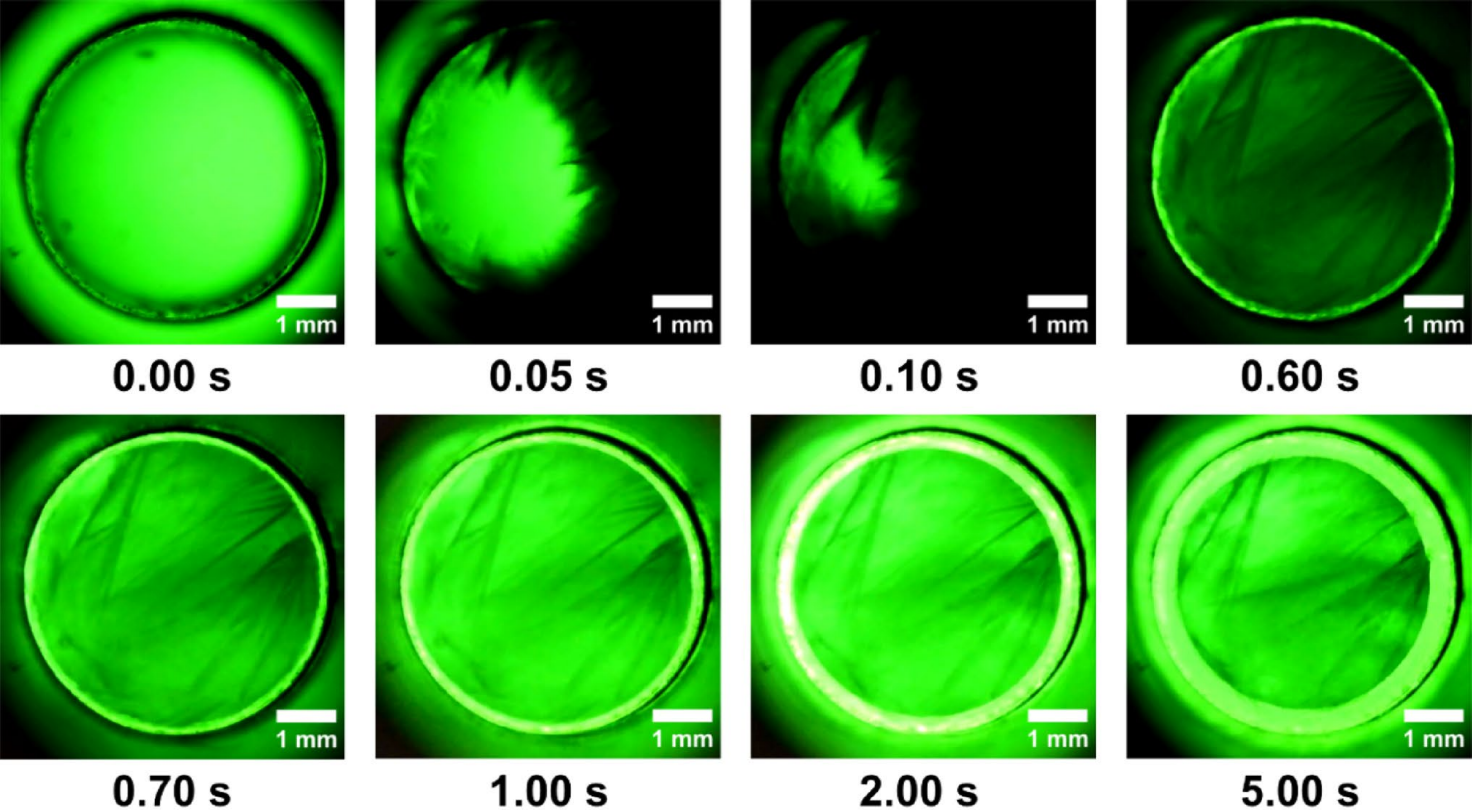
First 5 s after water nucleation into ice 1 h.

The isochoric reactor, which is cylindrical in shape and covered on top and bottom with sapphire glass covers, is located inside the device. With a 5 mm inner diameter and three different heights of 5 mm, 8 mm, or 10 mm, transform this device into a modular tool for the visual study of isochoric freezing phenomenon (Fig. 2). The ensure the safety of the experiments, the reactor was designed to withstand temperatures up to − 25 °C and pressure of 220 MPa^24^.

### Isochoric system—freezer, microscope

**Figure 3:**
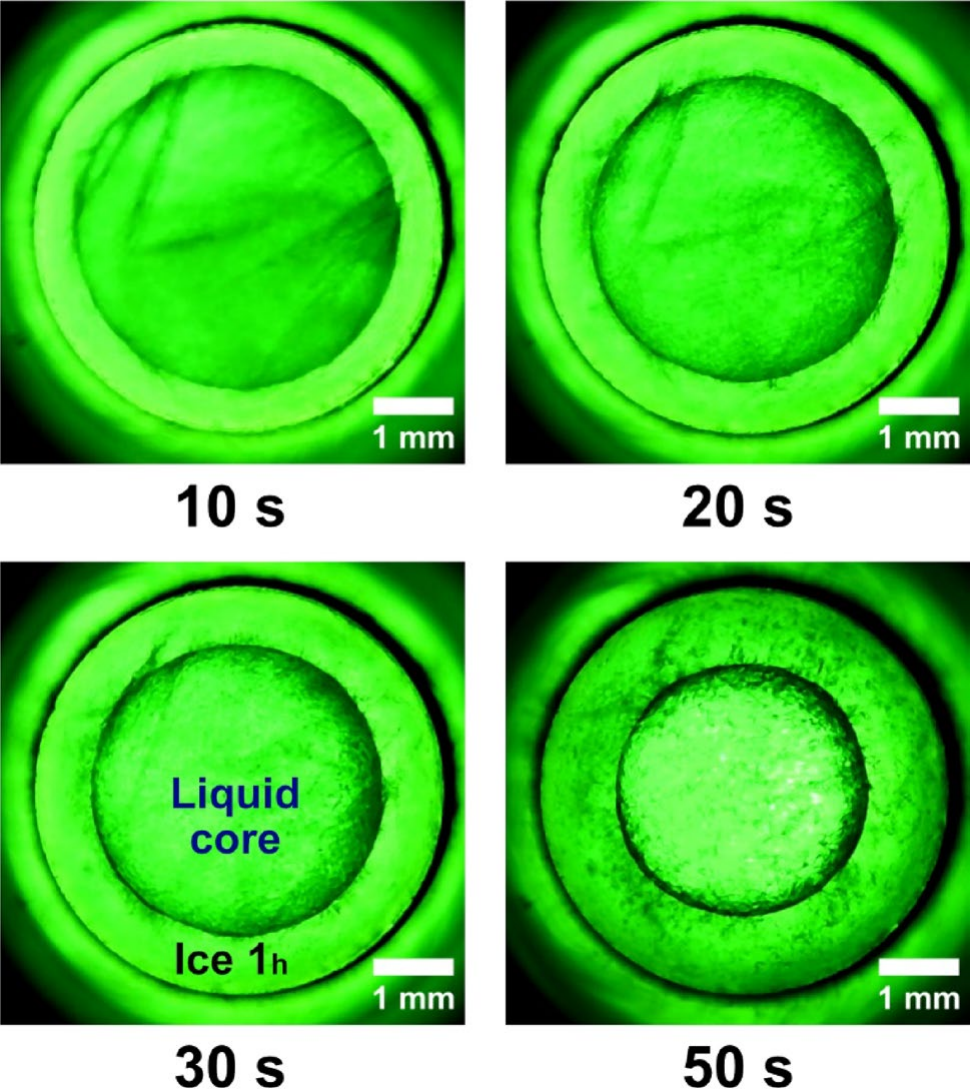
The early stages of ice crystal growth right after nucleation, the first 50 s. After the first 10 s the rate at which structural changes occur slows down.

**Figure 4:**
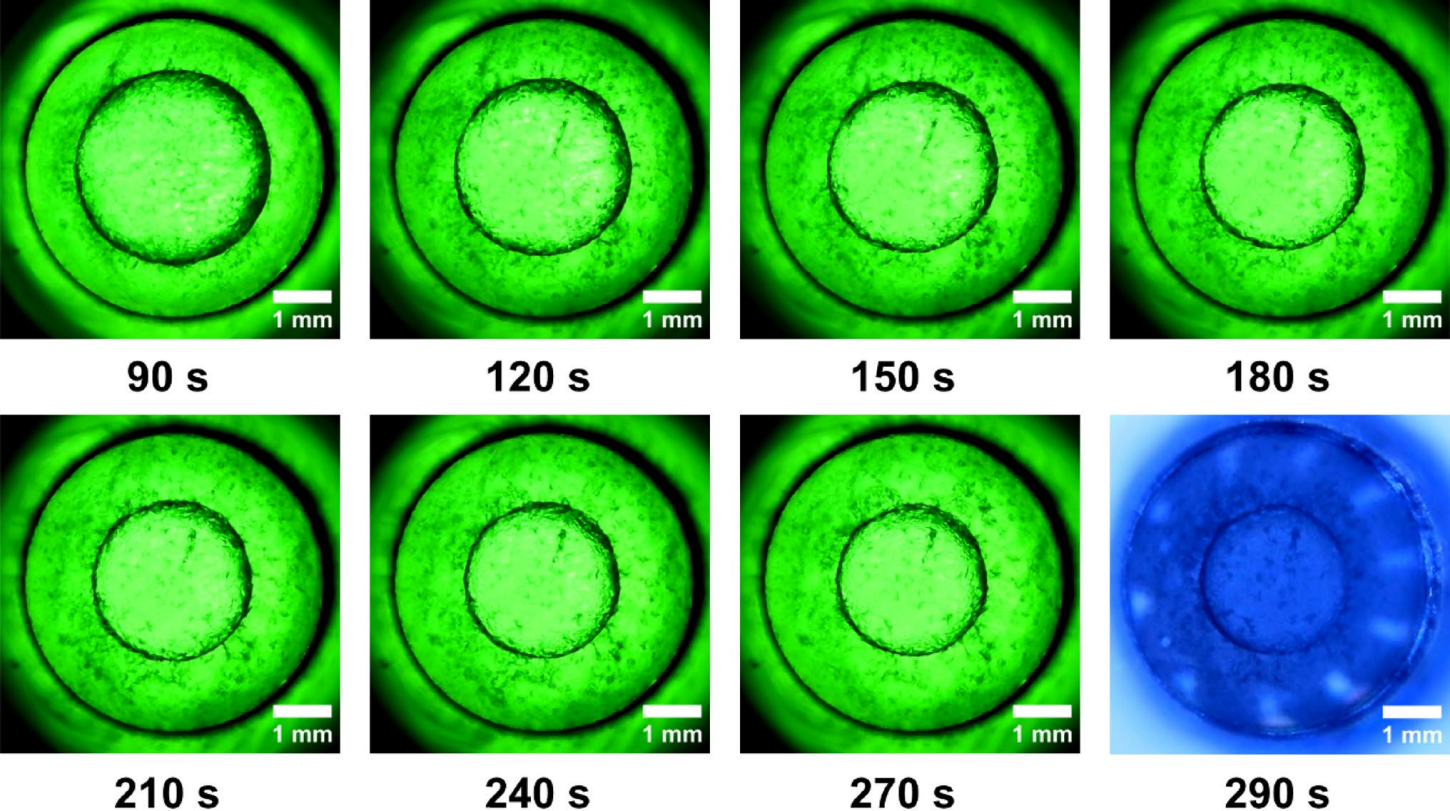
Almost 5 min after nucleation. The last picture is captured with the upper light, with a blue tint without colour filters. The white dots are the reflections of the LEDs that illuminate.

The cooling system which controls the temperature is formed from a cooling device, (RE 1225 S, Lauda Dr. R. Wobser Gmbh & Co. KG, Germany), which can lower the temperature to − 25 °C and uses a mixture of 50% ethylene glycol that is pumped through the low-temperature jacket of the isochoric reactor (Fig. 3). The temperature is measured on the inlet and outlet using two thermocouples (TL0024 PerfectPrime T-type (2 m long, specific for high accuracy measurements in the refrigeration and cryogenics field, with an excellent repeatability between − 200 °C and 260 °C), connected to a digital thermometer (MS6514 Mastech Digital Inc, China) which is linked to the laptop (ProBook 6 6570b, Hewlett Packard Enterprise, made in India) to display and record temperatures (Fig. 4).

**Figure 5:**
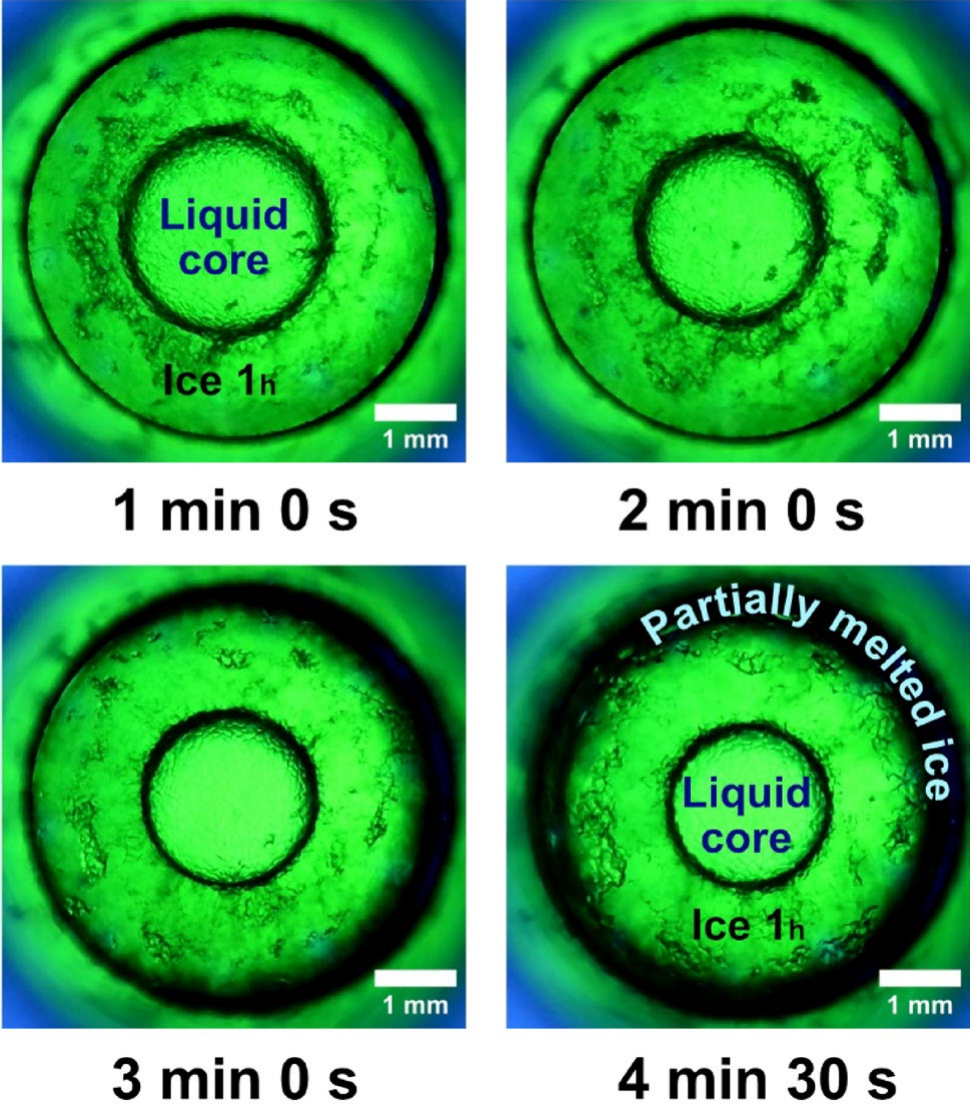
First 4 min and 30 s after the melting phase was initiated.

**Figure 6:**
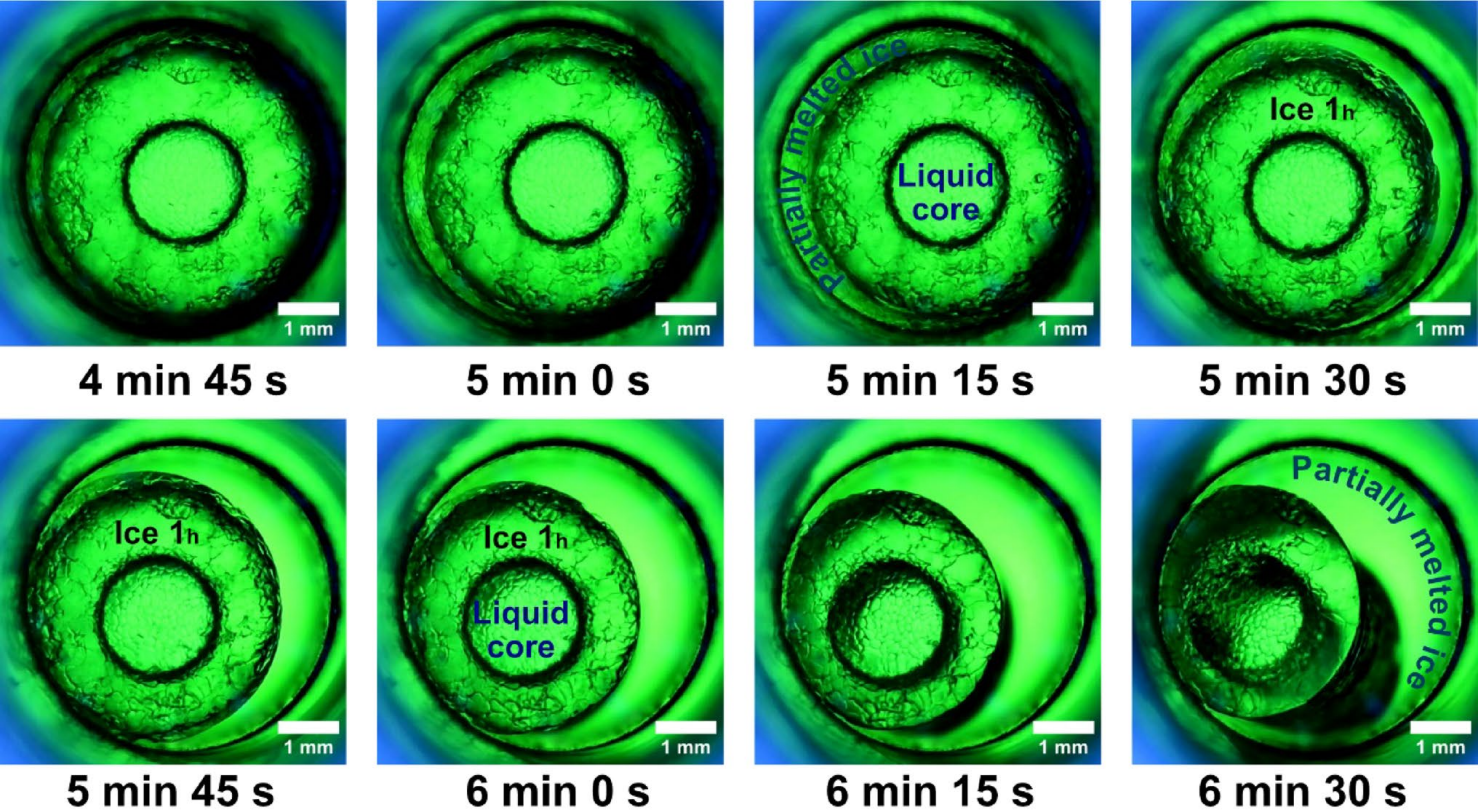
Thawing process in progress. Time is quantified from the moment it started the melting process.

**Figure 7:**
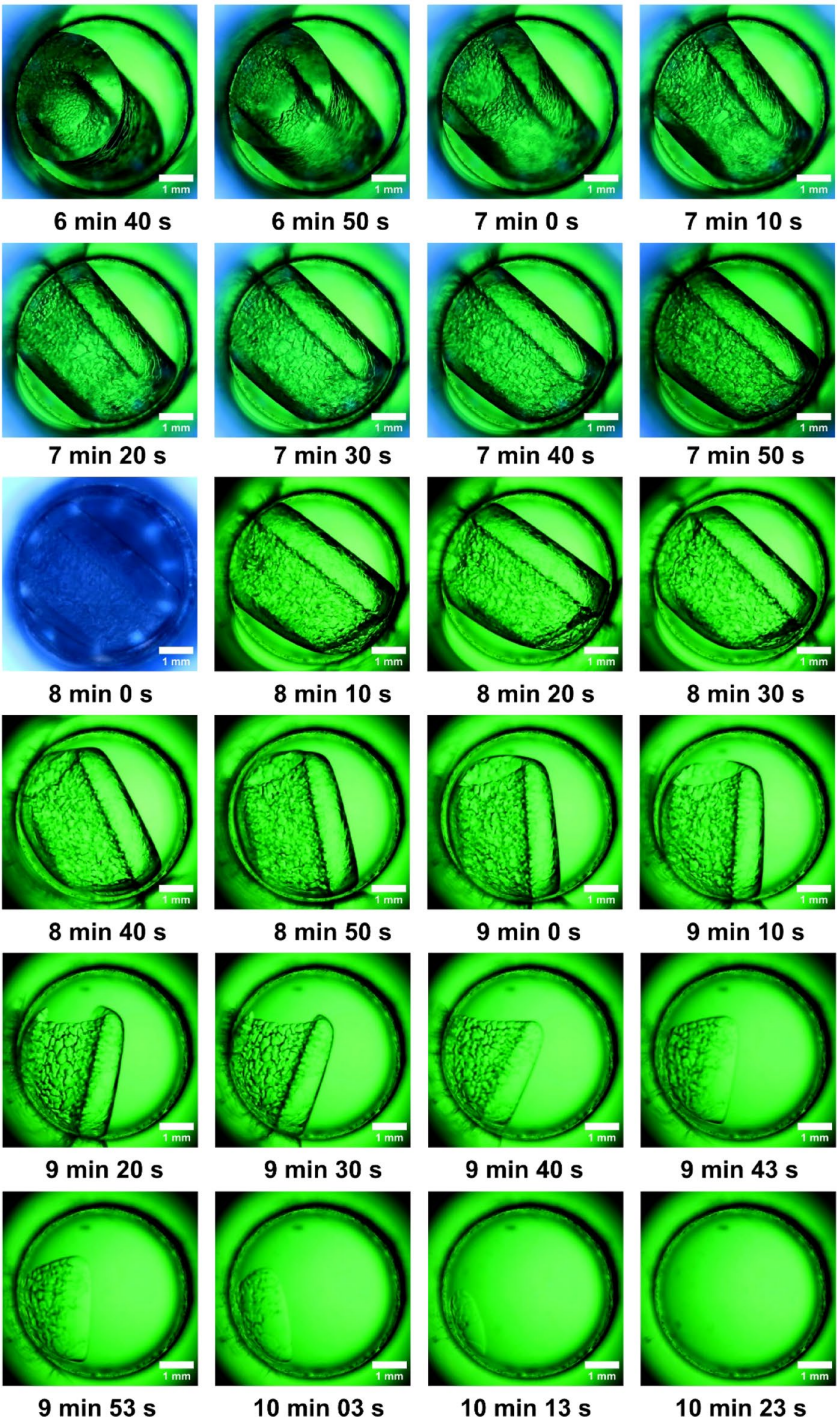
Thawing process on the final stage. The reactor’s entire liquid-ice mixture is brought back to its original state.

Since the study is visual, the issue of the ice core appearing directly on the transparent surface and making it opaque has been solved. However, the issue of condensation that may appear on the outer surface of the lens still exists (Fig. 5). As a thermal break, we used viewing windows made of acrylic plastic sheets that were attached to the exterior insulation (Fig. 6). Pressurized dry air spray for pc components (Air Duster Selecline, made in EU), was used to spray dry air into the orifice between the sapphire lenses and the acrylic plastic window in order to remove humidity and prevent condensation on the sapphire lenses^23^. Two computer fans have been used to enhance convection and prevent condensation on the surface as a final safeguard against issues with condensation on the protective acrylic plastic windows (Fig. 7).

**Figure 8:**
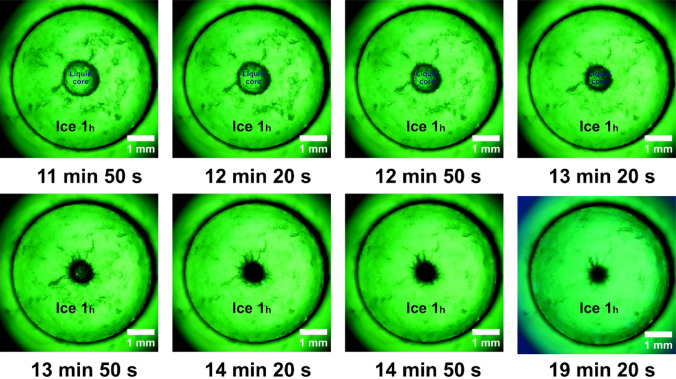
Ice crystals growth after nucleation phenomena in isochoric environment until thermodynamic equilibrium temperature measured at − 11.7 °C (− 13 °C actually setpoint on the cooling bath).

A digital microscope (WiFi Digital Microscope Easyover 315w, China) that was connected to an Android device allowed for the successful visualization of the interior of the reactor (Fig. 8). The highest resolution is up to five megapixels, the magnification level is up to × 500 with a lower and upper light source, a working height between 0–70 mm and focus range between 2 and 55 mm. It is also possible to capture both pictures and videos with this digital microscope, video is recorded at 30 FPS, (33.34 ms).

### The experimental protocol

To begin the tests, we first set up the equipment. The cooling bath is initially set to a temperature of 5 °C. The PC is linked to the datalogger that measures and logs the temperatures of the inlet and outlet from the isochoric reactor cooling connections, and the microscope is set up and connected to either the PC or an Android device. In the next step, the isochoric reactor is prepared for performing the experiments. Will choose the capacity of the inner volume with a height of 10 mm, and assemble the isochoric device as follows:

1. Placing the lower sapphire glass in its place.
2. The illustrated chamber in Fig. 1A was entirely filled with pure distilled water (Distilled water, European Drinks SA, RO) and sealed for isochoric conditions, taking care to avoid adding any bulk air bubbles, which can ruin isochoric conditions due to their high compressibility^25^.
3. By placing the upper sapphire glass with care, the isochoric volume is prepared for hermetically closing.
4. The whole assembly is stiffened and closed with the final threaded cover.
5. The lower and upper acrylic plastic sheets visualization screens are positioned accordingly with glue. To avoid condensation, dry air was poured into the space between them and the sapphire windows.

The inner chamber of the isochoric reactor is kept at the proper temperature by an ongoing flow across the cooling shell. Refrigeration bath water that has been combined with 50% ethylene glycol is continuously pumped through the apparatus. For precision, the inlet and outlet temperatures of the coolant are permanently measured and recorded using two T type thermocouples. To ensure a constant temperature of the sample, the entire apparatus is insulated with 9 mm elastomeric insulation.

We placed the isochoric reactor on the microscope, adjusted the focus and mounted a green colour filter over the lower light to create stronger contrast and highlight better the images inside the reactor, after which we started filming.

The experiments were conducted using a starting temperature of 5 °C and a cooling temperature setpoint of − 13 °C. The four temperatures that we anticipated to establish thermodynamic equilibrium were − 5 °C, − 6 °C, − 9 °C and − 13 °C. The state of equilibrium was verified by comparing the temperatures measured on the hose connections on inlet and outlet of the cooling fluid. The recorded cooling rate was on average − 18 °C/h. We know from previous isochoric experiments that water in confine spaces freezes randomly within the temperature range of − 7 °C to − 10°C^23,26^. This fact was also demonstrated visually in our experiments. Firstly, we chose an equilibrium threshold of a few minutes maintained at the − 6 °C for the visual capture of the supercooled state.

Once the nucleation occurs, the cooling continues until the final setpoint for each set of experiments. After a few minutes after the thermodynamic equilibrium is reached, a positive temperature, + 5 °C is set to visualize the melting phase. The melting is usually visible on the transparent windows after the system reaches + 2 °C.

Each experimental run was captured on camera. There were two freezing stages and two thawing phases in every session. For this particular work, ten of these repeats were conducted. Images were mostly taken using the lower portion of the microscope’s light through a green filter, although they were also taken using the upper portion of the light source.

### The scope of the experiments

With the set temperature close to − 13 °C, the first goal of the project was to observe the moments right after nucleation and then monitor the dynamics of the ice nuclei and growth in isochoric conditions. Seeing the opposite process of melting with all the moments leading up to the initial liquid phase was the second objective.

The aim of our study was to get authentic pictures and videos of this phenomenon, which has only been examined through other means until now, using opaque devices. We were successful in obtaining these, and the results will be shown in the following sections. The visual materials were then studied and interpreted in the results, discussions, and conclusions sections.

## Results and discussions

### The supercooled state of the pure distilled water under isochoric condition at subfreezing temperatures

The behaviour of deionized water in an isochoric environment at − 6 °C caught the interest of several trained investigations. We wanted to demonstrate in the experiment that follows that, visually stated, distilled water stays liquid (supercooling) below the − 6 °C barrier.

With the use of lower and upper lights, we were able to observe the supercooled state of water inside the reactor following thermodynamic balancing (the inlet temperature is equal to the outlet temperature) at the freezing setpoint of − 6 °C. When compared to the beginning of experiment, we saw no change in either form or structure. Again, this time, a visual demonstration of this occurrence was provided. At a temperature much below the freezing point, water remains liquid in isochoric conditions.

This was investigated and graphically shown previously in ISCM from the stability perspective^23^.

### Observation of the dynamic of the ice nucleation and ice growth in isochoric conditions—freezing

- (1) In Fig. 2 at 0.00 s depicts water in the isochoric supercooling condition at thermodynamic equilibrium, which was reached at a temperature of − 6 °C. The experiment was then conducted to − 9 °C. Before the nucleation started at about − 8.4 °C, the system was stable. The cooling rate was constant, on average − 18 °C/h. Random nucleation takes place extremely rapidly. The first 5 s after nucleation are shown in Fig. 2, and they are marked by incredibly quick shifts. Selected frames were obtained at a speed of 33 ms. In hundredths of a second, the microscope camera recorded very quick, various frames. It is important to note that the instant time = 0 s refers to the moment T_0_’ just prior to the nucleation, not the moment T_0_ when the reactor began to cool. The radial geometry of the reactor’s walls is replicated by the ice nucleus, as can be observed. The ice has a dendritic morphology, and it appears that the first 5 s of its development are like those in an isobaric environment^23^.

The following images were captured from the video recorded throughout the experiment.

- (2) At the beginning, the ice core is seen to burst rapidly from the reactor wall in a radial pattern; however, after the first 5 s, the total amount of ice development is suppressed. As a result, the liquid concentrates into a core nucleus. The type 1 h ice nucleus spreads at a significantly slower rate beyond the initial 10 s. This slowness was caused at this point by an increase in internal pressure that was getting close to 90 MPa. The container will apply an equal and opposite back pressure to the water and the ice core because the system’s overall volume remains constant. Due to this fact, the specific potential of water and ice is increased but their specific volume is decreased. We refer to this pressurization of the system and densification of the initial liquid phase as the “isochoric growth penalty”, which is the energetic cost incurred by the solid phase to grow within a system of limited absolute and specific volumes^28^. In Fig. 3 we have captured these phenomenon and is very clearly highlighted.
- (3) A stabilization of the typical water freezing process in an isochoric reactor is observed once the thermodynamic equilibrium is reached at a temperature of − 9 °C and 290 s after nucleation. The ice nucleus in the first 50 s after formation, begins to stabilize. The tendency is for the ice to advance towards the centre and force the liquid core to shrink. It is observed that there is no exact delimitation between ice and liquid, they are penetrated a little in the demarcation line. From the inner diameter of 5 mm of the reactor, the liquid core measures approximately 3.19 mm after 90 s. The ice advances and gradually concentrates the liquid nucleus up to 2.34 mm after 290 s from the beginning of nucleation. These values were measured on the images in Fig. 4. The values are approximations since the two aggregate states intersect.

### Observation of the dynamic of the development of ice crystals after nucleation in isochoric conditions—thawing

1. After the equilibrium state was confirmed at − 9 °C, the melting process was started. The temperature was set at + 5 °C, and the average heating rate was on average 60 °C/h. It should be noted that early evolution was slow due to thermal inertia, and even after heating has begun, the liquid core in the centre continues to have a tendency to constrict. The earliest indications of melting are seen on the outside of the ice layer (the dark-coloured borders) in Fig. 5.
2. After melting was initiated, the process accelerated, and photos were captured at 15 s intervals. It is observed how the warm exterior of the reactor melts the ice, and water begins to appear in liquid form. The ice layer shrinks, keeping its initial cylinder shape with a liquid core. Below, in Fig. 6 can be observed the dynamic of the process.
3. The thawing phase continues accelerated which is why in Fig. 7 we have exemplified a series of images captured with a step of 10 s. The ice layer is getting thinner and thinner. The ice cylinder rotates in the direction (left to right) of the heating agent’s flow due to the appearance of currents inside the reactor. Nearly all the melting has occurred. Different densities are present inside and outside the ice cylinder, as can be noticed visually. A liquid is floating around what's left of the cylindrical ice. We observe how a hole is well defined in the centre of the ice cylinder by the fluid core that has formed. The 8 min picture is captured with light from the upper part of the microscope, cool colour blue tint without colour filters. In the last images series of the Fig. 7 the thawing phase is done. The ice totally vanishes ten minutes after the melting process starts. The melting process began slowly and moved quickly to its finish. The photo frames were retrieved in an alternate sequence based on the internal dynamics and relevant images. The system reaches the same state as it had at the start of the freezing phase, with a clean image and no air bubbles that may have entered due to the vacuum at the end of the melting phase. It can observe the last ice formations that turn liquid.

### Dynamics of ice growth observed under isochoric conditions: prolonged freezing following nucleation

With a setpoint of − 13 °C, the freezing phenomenon is visualized in Fig. 8. The formation of a liquid core that resists the freezing phase is well observed. The ice grows in its occupied space as the temperature decreases. The diameter of the liquid core decreases and takes on an uneven shape with protrusions in the ice zone. Finally, it turned into a black dot in the cylinder’s centre. The system is thermodynamically balanced at the predetermined temperature of − 11.7 °C after more than 19 min, as confirmed by the cooling fluid’s inlet and outlet temperature equalization. This is the final moment before the thaw starts. We find a somewhat a slightly smaller nucleus, but with the same shape, see Fig. 8.

## Conclusions

### Shape and location

Following nucleation, when the water moves from its stage of aggregation into ice, the location and form of the forms of ice, water, and the ice-water mix are all determined by the internal shape of the reactor. The formation of cylindrical ice is clearly seen to occur from the outside in, with a liquid-mixed ice core concentrated inside. In terms of location, the ice originates at the reactor walls, which serve as a nucleation site. The reactor’s internal pressure rises as the ice spreads. As a result, a liquid core concentrates inside and the ice stops forming.

The details are crucial since they offer direction on where biological material should be safely stored for cryopreservation so that ice crystals won’t harm the tissue.

### Triggering the ice

Like how waves propagate in water, nucleation spreads in a circular motion. As we know from earlier research, the phenomena are extremely quick and strong, and there is an increase in internal pressure^26^. In this investigation, it was visually noticed that within the first 5 s, the effect is abrupt, swift, and violent (Fig. 2). As the pressure increases, the phenomenon slows down and prevents the formation of all ice crystals immediately after the first 10 s of the aggregation phase (Fig. 3). As the temperature continues to drop, the liquid circular core inside the reactor starts to constrict and the ice tends to take up more and more spate until the environment inside reaches the maturation and equilibrium phase. (Figs. 4 and 8). This dynamic is limited when the thermodynamic equilibrium is reached at the temperature of − 9 °C and respectively − 11.7 °C. Until now, alternative approaches have been used to forecast and investigate some of these observations. Being able to verify earlier findings with ocular observation was quite intriguing.

### Thawing phase

Melting, the opposing phenomenon, it was equally fascinating and unique. It was visualized immediately after the aggregation phase (Fig. 4). At the beginning, the dynamics is relatively slow due to thermal inertia. The melting gives us some spectacular images with liquid on the edges of the reactor, ice, and liquid inside the ice cylinder. As the thermal agent flows through the reactor mantle, the melting ice inside the reactor starts to rotate and float in that direction, producing images from multiple angles (Fig. 7). The water eventually becomes as clear as it was at the start of the experiment, with no evidence of the ice crystal remaining. Since an entire freeze–thaw cycle was conducted inside the isochore, this aspect of the experiment is considered as successful.

### Final reflections

This paper undertakes an experimental examination of water nucleation, employing a specially designed isochoric cylinder featuring transparent upper and lower sections. The utilization of an isochoric cylinder in this context facilitates a controlled and confined environment for the investigation, with the transparency of the vessel allowing for direct visual observation of nucleation phenomena throughout the experimental duration.

The study revealed a discernible prevalence of nucleation sites distributed heterogeneously across the confined water volume. These sites manifested as localized regions where the transition from the liquid to the nucleated phase was initiated. By employing advanced microscopic imaging techniques, the research successfully identified and characterized the spatial distribution of these nucleation sites. Initiation of the nucleation process is observed to originate proximate to the interior wall of the isochoric aluminium cylinder, following an anticipated pattern wherein nucleation events progress radially toward the central axis of the confined space. This outcome aligns with theoretical expectations, substantiating the influence of the container’s boundaries on nucleation site initiation and subsequent radial expansion. The observed nucleation and ice crystal dynamics conform to established principles governing phase transitions within confined environments, highlighting the spatially dependent nature of the nucleation phenomenon in the present experimental context.

The growth dynamics of nucleated structures were systematically examined, shedding light on the temporal evolution of nucleation events. The study discerned a distinct growth pattern, consisting of an anisotropic expansion, depending on the specific isochoric environmental conditions. The transparent design of the isochoric cylinder facilitated the direct observation of unique visual features associated with the water nucleation process. Intriguingly, the study identified visually distinct characteristics such as the formation of intricate dendritic structures, spatial heterogeneities in nucleation, and dynamic interactions between neighbouring nucleation events. These unique visual features not only enrich our qualitative understanding of nucleation but also provide a basis for further quantitative analyses, contributing to the refinement of theoretical models in the field. By delving into the specifics of nucleation sites, growth patterns, and unique visual features, this investigation has not only advanced our comprehension of isochoric water nucleation but has also laid the groundwork for refining theoretical frameworks and informing practical applications in diverse scientific and industrial domains.

The examination of temperature and pressure influences on water nucleation within the isochoric environment is a pivotal facet of this experimental inquiry. The isochoric system, characterized by a constant volume, provides a controlled setting to scrutinize the nuanced interplay between these thermodynamic variables. The experimental design allows for observations between cooling temperature, pressure generated, and the ice percentage formed from the total volume of liquid. Such results will be the subject of a new study, using the same isochoric device.

The temporal evolution of water nucleation within the confines of the isochoric cylinder constitutes a focal point of this experimental inquiry and consists of three distinct phases: the initial phase, early growth, and aggregation the second and maturation and equilibrium the last. The commencement of the temporal evolution is marked by the initiation of nucleation events near the interior surface of the isochoric cylinder. Analysis during this phase reveals insights into the role of heterogeneous nucleation sites and their spatial distribution along the container wall. Over time, the nucleation events exhibit discernible changes in size, shape, and spatial arrangement. The formation of intricate dendritic structures and the aggregation of nucleation sites contribute to the evolving landscape of the nucleation process. This phase sheds light on the interplay between molecular interactions, kinetic factors, and the evolving thermodynamic state within the isochoric system. As temporal progression unfolds, nucleation events reach a state of maturation characterized by stabilized sizes and configurations. This maturation phase signifies the attainment of equilibrium under the prevailing experimental conditions.

The practical implications of the study’s findings offer insights into controlling or manipulating nucleation processes for specific applications (biological matter preservation) or industries (food industry).

Future directions for future research based on the gaps or unanswered questions identified in our visual study could include exploring different experimental conditions, incorporating additional variables, or refining visualization techniques.

## Data Availability

The data used to support the findings of this study are available from the corresponding author upon request.

## References

[1] Bartel CJ (2018). Physical descriptor for the Gibbs energy of inorganic crystalline solids and temperature-dependent materials chemistry. Nat. Commun..

[2] Bore SL, Paesani F (2023). Realistic phase diagram of water from “first principles” data-driven quantum simulations. Nat. Commun..

[3] Pedersen UR, Costigliola L, Bailey NP, Schrøder TB, Dyre JC (2016). Thermodynamics of freezing and melting. Nat. Commun..

[4] Yamane R (2021). Experimental evidence for the existence of a second partially-ordered phase of ice VI. Nat. Commun..

[5] Mbah CF (2023). Early-stage bifurcation of crystallization in a sphere. Nat. Commun..

[6] Khan NS, Kumam P, Thounthong P (2020). Second law analysis with effects of Arrhenius activation energy and binary chemical reaction on nanofluid flow. Sci. Rep..

[7] Ooka H, Chiba Y, Nakamura R (2023). Thermodynamic principle to enhance enzymatic activity using the substrate affinity. Nat. Commun..

[8] Li C, Liu Z, Goonetilleke EC, Huang X (2021). Temperature-dependent kinetic pathways of heterogeneous ice nucleation competing between classical and non-classical nucleation. Nat. Commun..

[9] Fisher L, Gamble R, Middlehurst J (1981). The Kelvin equation and the capillary condensation of water. Nature.

[10] Hakimian A (2021). Freezing of few nanometers water droplets. Nat. Commun..

[11] Sun PZ (2021). Exponentially selective molecular sieving through angstrom pores. Nat. Commun..

[12] Powell-Palm MJ, Rubinsky B, Sun W (2020). Freezing water at constant volume and under confinement. Commun. Phys..

[13] Nastase G (2016). Advantages of isochoric freezing for food preservation: A preliminary analysis. Int. Commun. Heat Mass Transf..

[14] Li T, Donadio D, Galli G (2013). Ice nucleation at the nanoscale probes no man’s land of water. Nat. Commun..

[15] Powell-Palm MJ (2023). Cryopreservation and revival of Hawaiian stony corals using isochoric vitrification. Nat. Commun..

[16] Powell-Palm MJ (2021). Isochoric supercooled preservation and revival of human cardiac microtissues. Commun. Biol..

[17] Zhao Y, Lou L, Lyu C, Powell-Palm MJ, Rubinsky B (2022). Isochoric supercooling cryomicroscopy. Cryobiology.

[18] Takahashi KZ, Aoyagi T, Fukuda JI (2021). Multistep nucleation of anisotropic molecules. Nat. Commun..

[19] Loh ND (2017). Multistep nucleation of nanocrystals in aqueous solution. Nat. Chem..

[20] Karthika S, Radhakrishnan TK, Kalaichelvi P (2016). A Review of classical and nonclassical nucleation theories. Cryst. Growth Des..

[21] Bi Y, Cao B, Li T (2017). Enhanced heterogeneous ice nucleation by special surface geometry. Nat. Commun..

[22] Năstase G, Lyu C, Ukpai G, Şerban A, Rubinsky B (2017). Isochoric and isobaric freezing of fish muscle. Biochem. Biophys. Res. Commun..

[23] Zhao Y, Lou L, Lyu C, Powell-Palm MJ, Rubinsky B (2022). Isochoric supercooling cryomicroscopy. Cryobiology.

[24] Powell-Palm MJ, Rubinsky B, Sun W (2020). Freezing water at constant volume and under confinement. Commun. Phys..

[25] Perez PA, Preciado J, Carlson G, DeLonzor R, Rubinsky B (2016). The effect of undissolved air on isochoric freezing. Cryobiology.

[26] Câmpean SI (2021). Analysis of the relative supercooling enhancement of two emerging supercooling techniques. AIP Adv..

[27] Rubinsky B, Perez PA, Carlson ME (2005). The thermodynamic principles of isochoric cryopreservation. Cryobiology.

